# Editorial: Effects of physical exercise on brain and cognitive functioning, volume II

**DOI:** 10.3389/fnhum.2023.1360100

**Published:** 2024-01-09

**Authors:** Soledad Ballesteros, Laura Piccardi, Joshua Oon Soo Goh

**Affiliations:** ^1^Studies on Aging and Neurodegenerative Diseases Research Group, Department of Basic Psychology II, Universidad Nacional de Educación a Distancia, Madrid, Spain; ^2^Department of Psychology, Sapienza University of Rome, Rome, Italy; ^3^San Raffaele Cassino Hospital, Cassino, FR, Italy; ^4^Graduate Institute of Brain and Mind Sciences, National Taiwan University, Taipei, Taiwan

**Keywords:** active lifestyle, aging, cognitive functions, brain plasticity, physical exercise, young and older adults, wellbeing

Volume II of this Research Topic focused on the effects of physical activity on the brain and cognition. Physical activity is any body movement produced by skeletal muscles that requires energy expenditure. It is known that both moderate- and vigorous-intensity physical activity improve health (World Health Organization, 2018). Physical exercise is linked to the health state of the brain, affecting its cognitive functioning and plasticity. A growing body of research is highlighting how a physically active lifestyle is associated with reduced risk of dementia in old age, better cognitive functioning, physiological changes in the brain, and overall wellbeing. On the other hand, studies conducted on people with a sedentary lifestyle highlighted an increased risk of cardiovascular diseases and a higher rate of early mortality. Studies conducted in animals support this data by suggesting effects on neurotransmitter systems and neurotrophic factors, impacting synaptogenesis and neurogenesis.

Population aging will lead to demographic, personal, health, and social changes in the next decades. This situation will put a strain on the healthcare systems across the world, requiring more resources to attend to the needs of the large number of older adults in the coming years who will suffer cognitive decline and dementia. Promoting an active life and healthy aging is a priority considering the healthcare costs and the available resources (Persson et al., 2022). Not only in older adults, it is also crucial to maintain physical conditions and cognitive health throughout the lifespan.

Despite rapid growth in the field, many studies have yielded contrasting or inconclusive results due to variations in targeted populations, tests, and methodologies. Age and activity type are influencing brain state, but the mechanisms underlying it remain unclear. Recent research has explored the relationship between physical activity and cognitive functioning of the brain. This has helped to deepen our understanding of the correlations between these two factors. Additionally, the psychological benefits of leading an active lifestyle throughout the life span have also been addressed by these studies.

Our Research Topic includes four articles dealing with the effects of physical exercise and physical activity on the brain and cognition of young and older adults. It includes three original research articles and a brief research report.

Dong et al. used a cross-sectional study design to investigate the mediating effect of executive function between physical activity level and anxiety in a sample of 248 college students. The results showed that physical activity levels of college students were negatively associated with trait anxiety. An important finding of the study was that physical activity could indirectly affect trait anxiety by improving executive function. The study provides insight into the relationship between the level of physical activity, executive function, and emotion improvement mediated by executive function. Furthermore, the study informs of the behavioral mechanisms by which physical exercise improves mental health in young adults. Longitudinal studies should further investigate the correlation between physical activity levels, executive functions and trait anxiety. More research should directly evaluate physical activity levels instead of relying on self-reported questionnaires.

Several studies have shown the relevance of “expert-novice” paradigms in motor cognition investigations (e.g., Di Russo et al., 2005; Chang et al., 2011; Huang et al., 2018). In their study, Huang et al. used functional magnetic resonance imaging (fMRI) to investigate the neural mechanisms underlying decision-making of off-ball movements among football player experts and non-player college students, as well as the effects of long-term skill training on these neural mechanisms. In the study 20 professional college football players with an average training experience of 8 years (expert group) and 20 novice football players with no experience in sports disciplines (novice group). The authors investigated functional differences in brain activity in experts and non-experts when watching a video and when faced with an off-the-ball decision-making task. The study focused on the movement of players without a ball using moving imagery as a surrogate for motor execution. The expert participants showed higher accuracy than the control participants. The results showed significant disparities between players and non-players in brain activity in tasks involving motor video observation and decision-making. The results were consistent with the “neural efficiency hypothesis.” The novice group requires more brain function to process visual information compared to the expert group. Consistent with previous studies, both groups activated similar brain regions when viewing motion videos.

The brief research report by van der Sluys et al. examined the level of self-reported physical activity in a final sample of 50 young men (aged 18–27) experiencing multiple problems such as unemployment, substance use, history of delinquency, and lack of education. Their results were compared with that of an age and sex-matched control sample formed by 48 participants. Physical activity and cognitive control were assessed with the Physical Activity Questionnaire-Long Form (IPAQ), which measured the frequency, duration, and intensity of physical activity over the past 7 days. Three cognitive control tasks (Flanker, Go/NoGo, and Stroop) were used to assess cognitive control functions. Both groups showed similar activity levels, but the multi-problem group had impaired cognitive control, showed by decreased response inhibition and Flanker task performance compared to controls.

Many studies support the beneficial effects of being physically active in cognition and brain function in older adults. Piccardi et al. investigated the relationships between physical activity and memory complaints in attention, memory, and executive functions using a cross-sectional design. In a sample of 223 older adults without neurological or psychiatric disorders with a mean age of 74.84 years, members of Age Italia associations responded to a socio-demographic questionnaire and reported physical activity using a 3-point Likert scale (from 0 = never to 2 = often). Participants also completed a questionnaire to assess self-perceived working memory deficits in everyday life (WMQ) and a scale (DASS-21) to assess anxiety, depression, and stress. The findings showed that physical activity was associated with fewer attentional complaints and executive functions but not with memory storage. The results are in line with previous research and confirm that engaging in regular physical exercise produces a favorable effect on attention and executive functions, which depend on the frontal and prefrontal brain regions. The study's results suggested that regular physical activity is crucial for maintaining cognitive functions in older adults.

To conclude, these studies show that physical activity plays a central role in the brain and cognition of young and older adults. Future longitudinal intervention studies with experimental and active control groups will show types of exercises, training duration, and training intensity that are more successful in improving brain functioning, executive functions, and health conditions across the lifespan.

## Author contributions

SB: Writing—original draft, Writing—review & editing. LP: Writing—original draft, Writing—review & editing. JG: Writing—original draft, Writing—review & editing.
